# The experiences of patients who leave hospital against medical advice: analysis of survey data

**DOI:** 10.3389/frhs.2025.1620715

**Published:** 2025-09-26

**Authors:** Kyle Kemp, Brian Steele, Paul Fairie, Maria Jose Santana

**Affiliations:** ^1^ Department of Community Health Sciences, University of Calgary, Calgary, AB, Canada; ^2^ Patient Engagement Platform, Alberta Strategy for Patient-Oriented Research (SPOR), Calgary, AB, Canada; ^3^ Department of Paediatrics, University of Calgary, Calgary, AB, Canada

**Keywords:** survey, patient experience, leaving against medical advice (LAMA), hospital, patient-centered care

## Abstract

**Background:**

Historically, when patients leave hospital against medical advice (LAMA), the focus has often been on non-compliance or other patient-level factors, rather than on how services may be designed to better support these patients. Efforts to better understand why patients LAMA could strengthen the provision of patient-centered care that is responsive to individual needs and values. This study aimed to explore the experiences of Albertan adults who LAMA by examining patient-centered quality indicators (PC-QIs) derived from survey data. We sought to identify actionable insights that may inform service improvements and reduce the risks associated with LAMA discharges.

**Methods:**

We analyzed seven years of survey data, encompassing hospital discharges from April 2016 to March 2023. A random sample of respondents completed the Canadian Patient Experiences – Inpatient Care (CPES-IC) instrument by telephone within six weeks of hospital discharge. From the data, we assessed ten patient-centred quality indicators (PC-QI) which were previously co-created with patient advisors, researchers, and health system administrators. Survey responses/PC-QIs were reported as percent in “top box”, as represented by the most positive answer choice. Differences between patients who LAMA and other medical/surgical discharges were assessed.

**Results:**

A total of 144,480 surveys were successfully linked with inpatient records and included for analysis. This included 1,177 (0.9%) respondents who LAMA. In our sample, those who LAMA were predominantly male, younger, had a lower level of educational attainment, and were living with a greater number of comorbid health conditions. They also had lower self-reported levels of physical and mental health and had a longer average length of stay. The LAMA group had significantly lower top-box percentages on all ten of the PC-QIs which we examined. This difference ranged from 20.7% (communicating test results; 51.6% LAMA group vs. 71.3% others) to 29.2% (patient involvement in decisions about their care and treatment; 39.8% vs. 69.0% respectively).

**Conclusion:**

Patients who LAMA reported lower ratings of patient experience across all PC-QIs studied. Our findings may provide actionable, service-related insights into the reasons why patients LAMA. This is important as those who do so may place themselves at increased risk for future unplanned healthcare events, mortality, and morbidity.

## Introduction

Leaving hospital against medical advice (LAMA) occurs when patients choose to depart from a healthcare facility before the treating physician recommends discharge. Though often framed as a personal decision, LAMA discharges present complex clinical, ethical, and systemic challenges. In Canada, LAMA accounts for approximately 1%–2% of hospital discharges, with elevated rates among individuals with mental health conditions, substance use disorders, and those facing social vulnerabilities such as homelessness or poverty (1–4).

Patients who LAMA are at increased risk of adverse outcomes, including higher rates of morbidity, mortality, and unplanned readmissions (5, 6). These outcomes are often compounded by incomplete treatment, missed follow-up care, and disrupted continuity of care. In emergency department settings, patients who LAMA have been found to face up to four times higher readmission rates and increased healthcare costs, disproportionately affecting those with substance use disorders, mental illnesses, and low-income backgrounds (7).

While quantitative studies have identified correlates of LAMA, such as younger age, male sex, and diagnoses related to substance use, survey-based insights into patient experiences and motivations remain limited in the Canadian context. International literature highlights common reasons for LAMA departure, including disagreement with treatment plans, long wait times, perceived recovery, family obligations, financial constraints, and dissatisfaction with care (8). In Alberta, a mixed-methods study found that First Nations patients were more likely to leave care prematurely, often citing racism, stereotyping, poor communication, and systemic barriers such as transportation challenges and overcrowding (9).

Emerging evidence suggests that decisions to LAMA are frequently driven by systemic and interpersonal factors, including poor communication, perceived stigma, and unmet psychosocial needs (10, 11). These experiences suggest that LAMA discharges are often symptoms of unmet needs within the healthcare encounter rather than acts of defiance. For marginalized populations, such as Indigenous patients or people who use illicit drugs, experiences of discrimination and mistrust further contribute to premature departures from care.

Historically, patients who LAMA have been labeled as non-compliant, with limited attention to how health systems might better support them. However, a shift toward patient-centered care – with a focus on responsiveness to individual needs, values, and preferences, offers a more constructive lens. Reframing LAMA as “premature discharge” and adopting a shared responsibility model between patients and healthcare systems may help identify service-level gaps and improve care quality.

Given the gaps in knowledge pertaining to the experiences of the those who choose to LAMA, the present study aimed to explore the experiences of Albertan adults who LAMA, using secondary analysis of responses to the Canadian Patient Experiences Survey – Inpatient Care (CPES-IC). By examining patient-centered quality indicators (PC-QIs), co-developed with patients and clinicians, we sought to identify actionable insights that may inform service improvements and reduce the risks associated with LAMA discharges. Understanding these experiences is essential for designing respectful, effective strategies that promote continuity of care and reduce the frequency and consequences of LAMA events.

## Methods

### Study design and setting

This study was a retrospective analysis data collected over a seven-year period from April 2016 to March 2023 in the province of Alberta, Canada. Over this time, Alberta Health Services (AHS) was the sole provider of acute care services across the province, and was Canada's largest province-wide, integrated healthcare system. Each year, AHS delivers care in over 400,000 hospital stays, resulting in over 2.9 million hospital bed days (12). An AHS key strategy which aligns with the spirit of this study is the “Patient First” one, which aims to promote respectful patient/provider interactions, improve communication between providers and patients/families, to adopt a team-based approach to care, and improve transitions in care. Alberta was selected as the study location because the research team had access to comprehensive, linked administrative and survey data from Alberta Health Services (AHS), the sole provider of acute care services in the province. The sample included all hospitals in Alberta with overnight inpatient services (*n* = 93), encompassing both generalist and specialized facilities.

Ethics approval to conduct the study was obtained from the University of Calgary Conjoint Health Research Ethics Board (CHREB), with a waiver of patient consent granted. De-identified, linked record-level data were provided by AHS under a research agreement, with all linkages performed by AHS staff.

### Data sources

Between two days and six weeks following their hospital discharge, a random sample of patients was contacted by AHS to complete a customized version of the Canadian Patient Experiences Survey – Inpatient Care (CPES-IC) instrument (13, 14). Using a standardized script, prompts, and answers to frequently asked questions, consenting respondents were asked 56 questions regarding their hospital experiences. Historically, the survey has taken respondents approximately 15 min to complete. Since its introduction, the response rate has been in the 60% range, with AHS capturing approximately 25,000 completed surveys each year. Contact with AHS was established via telephone, using a standardized outreach protocol. The survey was administered through computer-assisted telephone interviewing (CATI). No follow-up actions were employed. Participants were informed about the voluntary nature and anonymity of the study, and verbal consent was obtained prior to participation.

CPES-IC topics cover multiple domains of inpatient care, including admission to hospital, emergency department experiences (as applicable), communication with nurses and physicians, the hospital environment, pain management and medications, information sharing, patient/family involvement, discharge planning, and concerns regarding care. Except for two open-ended questions, responses were captured using a Likert scale (e.g., always, usually, sometimes, never). The most positive option to each question was classified as a “top box” response, in alignment with the Hospital Consumer Assessment of Healthcare Providers and Systems (HCAHPS) survey (15, 16), which was the basis for the CPES-IC tool. CPES-IC survey data were mapped to a set of patient-centered quality indicators (PC-QIs) which were co-created by Santana et al. with patient and clinician input (17, 18). Missing data were handled by excluding incomplete responses from the analysis.

Completed surveys were linked to its corresponding clinical record in the Discharge Abstract Database (DAD) (19), which includes all inpatient discharge records across Alberta. Individual linkage was done by AHS using an exact match on personal health number/unique lifetime identifier (ULI), hospital code, and discharge date.

### Study population

Adult patients (18 years or older at time of hospital discharge) who could independently respond to the CPES-IC were eligible to complete the survey. Complete survey eligibility criteria have been published elsewhere (15, 20). For the purposes of analysis, respondents were classified into two groups. The LAMA group was comprised of all CPES-IC respondents with a discharge disposition code of “06”, “61”, “62”, or “65” in the DAD. The comparison group was comprised of all other discharges where the respondent was discharged home (disposition code of “05”) (21).

### Statistical analysis

Differences in demographic and clinical characteristics between the two study groups were assessed using chi-square analyses. All values of *p* < 0.05 were considered statistically significant. Demographic variables included sex, age group (at time of discharge: 18–50 years, 51–64, 65–74, 75 years and older), level of education (high school or less, college or undergraduate university, post-graduate or professional degree), and self-rated mental and physical health (each reported as excellent, very good, good, fair, poor). Clinical variables included number of medical comorbidities (none, 1, 2 or more), admission type (urgent, elective), hospital type (large urban, other), admission category (urgent, elective), number of Elixhauser comorbidities (20), and length of hospital stay (less than 3 days, 3–7, greater than 7 days).

PC-QIs were calculated as percent in “top box” for each study group, where “top box” corresponded to the most positive answer choice (16). In cases where multiple survey questions aligned with a given PC-QI, the average percentage was reported for the given PC-QIs. All analyses were done using SAS 9.4 for Windows.

## Results

Over the seven-year study period, a total of 144,480 completed surveys were linked with its corresponding inpatient record. This number included 1,177 in the LAMA group (0.8% of total). Demographic and clinical characteristics are presented in Table 1 for both groups. From a demographic perspective, the LAMA group had higher proportion of males, was younger, had lower levels of educational attainment, and had worse self-reported physical and mental health (*p* < 0.001 for all comparisons). Clinically, the LAMA group had more documented medical comorbidities, were admitted to hospital mostly on an urgent basis, were seen less in large urban hospitals, and had longer lengths of stay (*p* < 0.001 for all comparisons). This table provides a detailed profile of the hospitalized patients, including sex, age, education level, comorbidities, admission type, hospital type, length of stay, and self-reported physical and mental health.

**Table 1:** Characteristics of the sample.

Variable	LAMA (*n* = 1,177)	Discharged home (*n* = 143,303)	*p*
	*n* (%)	*n* (%)	
Sex			
Female	599 (50.9)	91,334 (63.7)	<.001
Male		578 (49.1)	51,969 (36.3)
Age group			
18–50 years	551 (46.8)	65,534 (44.3)	<.001
51–64 years	352 (29.9)	32,958 (23.0)	
65–74 years	184 (15.6)	27,643 (19.3)	
75 years or older	90 (7.7)	19,166 (13.4)	
Education level (*n* = 140,425)			
High school or less	618 (54.5)	53,703 (38.6)	<.001
College or undergraduate university	426 (37.6)	68,003 (48.8)	
Post-graduate/professional degree	90 (7.9)	17,585 (12.6)	
Number of medical comorbidities			
None	616 (52.3)	100,369 (70.0)	<.001
1	341 (29.0)	28,785 (20.1)	
2 or more	220 (18.7)	14,149 (9.9)	
Admission type			
Urgent	1,009 (85.7)	74,222 (51.8)	<.001
Elective	168 (14.3)	69,081 (48.2)	
Hospital type			
Large urban	749 (63.6)	114,551 (79.9)	<.001
Other	428 (36.4)	28,752 (20.1)	
Length of hospital stay			
Less than 3 days	501 (42.6)	70,990 (49.1)	<.001
3–7 days	460 (39.1)	53,329 (36.9)	
Longer than 7 days	216 (18.4)	18,984 (13.1)	
Self-reported physical health (*n* = 143,282)			
Excellent	102 (8.8)	23,694 (16.7)	<.001
Very good	209 (17.9)	47,217 (33.2)	
Good	347 (29.8)	41,815 (29.4)	
Fair	293 (25.1)	20,683 (14.6)	
Poor	215 (18.4)	8,707 (6.1)	
Self-reported mental health (*n* = 143,785)			
Excellent	164 (14.1)	37,921 (26.6)	<.001
Very good	280 (24.1)	50,309 (35.3)	
Good	345 (29.7)	38,141 (26.7)	
Fair	228 (19.6)	12,782 (9.0)	
Poor	145 (12.5)	3,470 (2.4)	

Table 2 presents the “top box” percentages for each of the ten PC-QIs, for both the LAMA and comparison groups. Among respondents who LAMA, patient involvement in decisions about their care and treatment was the lowest scoring PC-QI (39.8% in “top box”). Although still quite low, the highest scoring PC-QI was communication between patient and nurses (56.0% in “top box”). When compared with those discharged home, respondents in the LAMA group reported lower scores on all ten PC-QIs which we studied. The gap between the two groups ranged from 20.7% (communicating test results) to 29.2% (patient involvement in decisions about their care and treatment). The LAMA group had 35.8% reporting a “top box” score for their overall rating of care. This represented a difference of 26.8 percentage points from the comparison group (62.6%). All observed differences between the two groups in Table 2 were statistically significant (*p* < 0.001).

**Table 2:** Survey results; by PC-QI.

PC-QI	Percent in “Top Box”		Percent difference	*p*
	LAMA	Discharged home		
Overall rating	35.8	62.6	26.8	<.001
Family and friends test	45.0	72.8	27.8	<.001
Communication between patient and nurses	56.0	78.7	22.7	<.001
Communication between patient and physicians	53.2	79.7	26.5	<.001
Information about taking medications	48.9	75.3	26.4	<.001
Communicating test results	51.6	72.3	20.7	<.001
Coordination of care	46.7	68.8	22.1	<.001
Patient involvement in decisions about their care and treatment	39.8	69.0	29.2	<.001
Engaging patients in managing their own health	49.5	70.9	21.4	<.001
Transition planning	50.3	76.6	26.3	<.001

## Discussion

Patients who LAMA present significant challenges for both healthcare systems and patient outcomes. In our study, we showed that patients who LAMA report much lower ratings of patient experience. This was universally seen across all ten PC-QIs which were studied, demonstrating that these poor experiences transcend multiple aspects of care. Understanding patients' experiences reveals that LAMA discharges are often not impulsive decisions but reflect deeper issues within the hospital environment and the patient-provider relationship.

Our results are congruent with one of the most consistently reported themes in the literature - the role of poor communication. In addition to the studies referenced in the introduction, international works have shown that patients frequently cite feelings of being misunderstood, dismissed, or inadequately informed about their care plans as major factors influencing their decision to LAMA (22–24). A lack of shared decision-making and rigid hospital processes can exacerbate feelings of disempowerment among patients, leading to frustration and mistrust. These experiences suggest that LAMA discharges are often symptoms of unmet needs within the healthcare encounter rather than acts of defiance.

Evidence-based strategies to reduce LAMA have emerged from both Canadian and international literature. One such strategy is patient-centred communication and shared decision-making. By exploring reasons for wanting to leave, using non-stigmatizing language, and co-creating care plans, providers can reduce the risk of LAMA discharges (25, 26).

Beyond communication issues, external pressures play a crucial role. Many patients report LAMA due to obligations outside the hospital, such as employment, caregiving responsibilities, or financial constraints. In such cases, the hospital's inability to accommodate these external realities contributes to patients feeling that remaining hospitalized is untenable. Another important factor is perceived stigma—particularly among populations with substance use disorders or mental health conditions. Studies indicate that these patients often feel judged by healthcare staff, which in turn fuels decisions to LAMA (27). The emotional toll of perceived discrimination further alienates patients from the system that is supposed to support them. Importantly, patients' emotional experiences around LAMA discharges are complex. While some feel relief or empowerment at reclaiming autonomy, many later express regret or acknowledge that their health suffered as a result of leaving prematurely. This duality highlights that decisions to leave LAMA are often conflicted and fraught with anxiety rather than clear-cut acts of self-determination. Early identification and treatment of withdrawal and substance-related needs is another critical intervention. Rapid initiation or continuation of opioid agonist therapy, nicotine replacement, and timely addiction consults have been shown to decrease LAMA rates (28).

To address the most deficient patient-centered quality indicators (PC-QIs), particularly shared decision-making, evidence-based strategies such as the teach-back method, structured discharge planning tools, and culturally responsive care models have shown promise in reducing instances of leaving against medical advice (LAMA). The teach-back method, which involves asking patients to repeat back information in their own words, ensures comprehension and fosters collaborative communication—an essential component of shared decision-making (29). Structured discharge planning tools, including individualized care plans and early involvement of multidisciplinary teams, have been associated with improved patient satisfaction and reduced premature discharges. Furthermore, embedding culturally safe practices, such as employing liaison officers or health workers from the patient's community, can build trust, particularly among equity-seeking populations, and mitigate systemic barriers that contribute to LAMA. These interventions not only enhance communication and care coordination but also align with the principles of learning health systems by promoting continuous feedback and patient engagement.

Sadly, there may still be a tendency among some healthcare providers to frame LAMA solely as patient noncompliance – which may hinder opportunities for meaningful intervention. Recent work by Ambasta et al. has challenged the view that patients who LAMA are merely exhibiting deviant behavior. The authors argue that such discharges often reflect systemic failures in healthcare delivery, particularly in providing patient-centered care. As such, the authors advocate for reframing LAMA as “premature discharge” and suggest adopting a shared responsibility model between patients and healthcare systems (11). This approach emphasizes the need for healthcare systems to critically analyze each instance to improve care quality and better serve patients.

As with any study, ours has notable limitations. With surveys such as the CPES-IC, there is always the potential for recall bias. Secondly, it is plausible that many patients who LAMA may have been so disenchanted with their care, that they may have refused to complete the survey if telephoned. We were unable to obtain response rates according to discharge disposition to substantiate this possibility. However, our overall sample was comprised of 0.9% LAMA discharges, which is close to the 1%–2% reported in previous literature. With the survey protocol, the CPES-IC is administered in English only in Alberta, does not sample patients who were hospitalized due to a mental health concern, and does not allow for proxy respondents. Given these, it is plausible that some patients who LAMA were excluded from participating, which may impact the generalizability of our findings. While education level was available and included in our analysis, data on socio-economic status and specific types of illnesses were not available in the linked dataset. This limits our ability to fully characterize the social determinants of health and clinical diagnoses that may influence LAMA decisions.

In conclusion, our findings support the notion that interventions aimed at reducing instances of LAMA should focus not only on patient education but also on improving provider communication, promoting patient-centered care, and addressing systemic barriers. More inclusive, flexible, and empathetic approaches could help bridge the gap between patients’ needs and institutional policies, potentially reducing the frequency and adverse outcomes of LAMA discharges. Admittedly, it was disappointing to see that differences in patient experience results was not limited to one or two topics, which limits the actionability of our findings. We advocate that other approaches such as analysis of patient comments may provide more tailored insights for actionable improvements in the care for patients who may LAMA across our province or elsewhere.

## Data Availability

The data analyzed in this study is subject to the following licenses/restrictions: Data are identifiable and are under the custodianship of Alberta Health Services. All data for the study were linked by the health authority and provided to the team in linked, de-identified fashion. Requests to access these datasets should be directed to Jeff Bakal, research.administration@ahs.ca.
